# Drug-induced psychosis and intravenous drug use in chemsex context

**DOI:** 10.1192/j.eurpsy.2024.852

**Published:** 2024-08-27

**Authors:** J. Curto Ramos, A. Rodríguez Laguna, P. Barrio, L. Ibarguchi, A. García, I. Azqueta, H. Dolengevich Segal

**Affiliations:** ^1^Department of Psychiatry, Clinical Psychology and Mental Health, La Paz University Hospital; ^2^ Apoyo Positivo; ^3^Dual Disorders Program. Department of Psychiatry, Henares University Hospital, Madrid, Spain

## Abstract

**Introduction:**

Several studies have called atention to the mental health disorders associated with chemsex -the intentional use of drugs before or during sexual intercourse GBMSM (gay, bisexual and men who have sex with men) population-. Sexualized intravenous drug use is also known as slam or slamsex. There are few studies that analyze the mental health differences between intravenous drug users compared to non-intravenous drug users in chemsex context.

**Objectives:**

We aim to analyze the relationship between the practice of slamsex and the development of drug-induced psychosis.

**Methods:**

A cross-sectional descriptive analysis of a sample of users attended by the non-governmental organization Apoyo Positivo in the program “Sex, Drugs and You” between 2016-2019 was performed.

**Results:**

We included 217 participants. Drug-induced psychosis was found in 80 participants. Drug-induced psychosis was significantly higher in the intravenous drug use group compared to the non-intravenous drug use group (p<0.05).

**Conclusions:**

Previous studies have reported that MSM who practiced chemsex were more likely to experience from different mental health disorders, being psychosis one of the most frequent psychiatric diagnoses. In our study, drug-induced psychosis was higher in participants who engaged in intravenous drug use. Further studies analyzing the relationship between slamsex and drug-induced psychosis are needed.

**Disclosure of Interest:**

None Declared

